# ECT practice in England from 2012/13 to 2018/19: a retrospective analysis

**DOI:** 10.1192/bjo.2021.673

**Published:** 2021-06-18

**Authors:** Mohan Gondhalekar, Robert Chaplin, Sinead Rogers, Oriana Delgado

**Affiliations:** 1 Royal College of Psychiatrists, Consultant Old Age Psychiatrist and Clinical Fellow, Older Adult Mental Health Team Auckland District Health Board; 2 Royal College of Psychiatrists, Research Fellow and Consultant General Adult Psychiatrist; 3 Deputy Programme Manager ECTAS CCQI RCPsych; 4 Royal College of Psychiatrists

## Abstract

**Aims:**

The purpose of this study was to look longitudinally at ECT practice in England over the past 7 years: namely over the following key time periods; 2012/13, 2014/15, 2016/17, 2017/18, and 2018/19. A previous study by Chaplin et al, published in 2016, found that there had been a striking decline observed in the number of courses of ECT prescribed to patients from 2006 to two time points i.e. 2012/13 and 2014/15.

In this study we investigated whether or not this trend had continued. Hence we looked at the change in frequency of ECT use, the length of ECT courses, patient demographics and clinical outcomes; between 2012/13 and 2018/19.

**Background:**

Electroconvulsive therapy (ECT) is an effective treatment for Major Depression, Treatment-Resistant Depression, Catatonia, and Clozapine-resistant psychosis. There have been regular improvements in the administration of ECT, over the past two decades. Increases in the volume of the hippocampus and the amygdala have consistently been observed in ECT studies. Stigma has been the major barrier to patients receiving ECT in a timely fashion. The Royal College of Psychiatrists (RCPsych) Centre for Quality Improvement (CCQI) established the ECT Accreditation Service (ECTAS) back in 2006. ECTAS had the aim of standardising ECT practice through the production of evidence-based standards that all member ECT Clinics could use to support their practice.

**Method:**

We looked at the minimum dataset of information collected from ECTAS Members within England for the following years; 2012/13, 2014/15, 2016/17, 2017/18, 2018/19. In 2012/13, 2325 adjusted courses of ECT treatment were given to patients in England. In 2014/15 it was 2302.

**Result:**

Between 2012/13 and 2018/19; two thirds of ECT patients continue to be female. The modal age of patients has also remained the same at 70 years. The number of patients detained under the Mental Health Act 1983 receiving ECT has gone up by 12%; suggesting that the patients receiving ECT were more clinically unwell. After treatment, CGI scale scores (i.e. the very much improved and much improved scores) slightly reduced by 6% from 2012/3 to 2018/19.

**Conclusion:**

The use of ECT in England notably declined from 2006 to 2012/13 and 2014/15. However, from 2012/13 to 2018/19, ECT use has remained relatively stable; suggesting that it is currently being used appropriately on patients, who are amongst the most severely unwell. The clinical effectiveness of ECT remains high however, it has slightly dipped by 6%.

